# Impact of Prehospital Blood Pressure Profile on Functional Outcome After Traumatic Brain Injury

**DOI:** 10.3390/jcm14248808

**Published:** 2025-12-12

**Authors:** Daniel Ahlert, Giovanna Brandi, Alexander Kaserer, Adjmal Mirbaz, Roman Pfeifer, Alberto Pagnamenta, Simone Unseld

**Affiliations:** 1 Institute of Intensive Care Medicine, University Hospital Zurich, Rämistrasse 100, 8091 Zurich, Switzerland; daniel.ahlert@usz.ch (D.A.); giovanna.brandi@usz.ch (G.B.); adjmal.mirbaz@usz.ch (A.M.); 2 Department of Cardiology, University Hospital Zurich, Rämistrasse 100, 8091 Zurich, Switzerland; 3 Institute of Anesthesiology and Perioperative Medicine, University Hospital Zurich, University of Zurich, 8091 Zurich, Switzerland; alexander.kaserer@usz.ch; 4 Department of Trauma Surgery, University Hospital Zurich, Rämistrasse 100, 8091 Zurich, Switzerland; roman.pfeifer@usz.ch; 5 Department of Intensive Care, Civic Hospital Lugano, Ente Ospedaliero Cantonale, Via Tesserete 46, 6900 Lugano, Switzerland; alberto.pagnamenta@eoc.ch; 6 Clinical Trial Unit, Ente Ospedaliero Cantonale, 6900 Lugano, Switzerland; 7 Division of Pneumology, University Hospital of Geneva, 1205 Geneva, Switzerland

**Keywords:** traumatic brain injury, prehospital emergency care, systolic blood pressure, prognosis

## Abstract

**Background/Objectives**: Prehospital management after traumatic brain injury (TBI) focuses on the avoidance of secondary injuries such as derangement of blood pressure. Recent guidelines specify an updated optimal systolic blood pressure (SBP) target of 110–149 mmHg. We aim to characterise the prehospital blood pressure profile of patients including the SBP range and variability after TBI, amongst other prehospital parameters, to determine associations with the outcome. **Methods**: We performed a retrospective cohort study of adult patients admitted to the intensive care unit at University Hospital Zurich. The first recorded SBP, SBP variability, and average range during two-thirds of the prehospital time were analysed along with other prehospital parameters for survival and GOSE at hospital discharge using univariate and multivariable logistic regression analyses. **Results**: In total, 680 patients were included, of whom 76% had moderate to severe head injury and 117 patients died. Among the sample, 51% of patients were in the target range of 110–149 on initial assessment and 50% remained in this range during 2/3 of the prehospital time. The initial SBP, SBP variability, and SBP range were significant for survival in the univariate analysis, but they lost statistical significance in the multivariable model. This may indicate a reduced effect of the analysed SBP parameters on the outcome once controlling for confounding factors. In the multivariable analysis, catecholamine administration reduced the odds of an unfavourable GOSE at hospital discharge (OR 1.84 [1.20–2.81], *p* = 0.005), which may point towards a benefit of early haemodynamic stabilisation after injury. A younger age (OR 0.95 [95% CI 0.93–0.97], *p* < 0.001), lower AIS Head/Neck (OR 0.45 [0.29–0.70], *p* < 0.001), higher initial GCS (OR 1.24 [1.15–1.35], *p* < 0.001), and higher first haemoglobin (OR 1.24 [1.04–1.46], *p* = 0.014) were independent predictors of survival. **Conclusions**: Haemodynamic instability in the prehospital phase is common after TBI and represents a potentially modifiable factor. Catecholamine administration was associated with improved functional recovery, suggesting a possible role of prehospital haemodynamic management, although causality cannot be inferred.

## 1. Introduction

Traumatic brain injury (TBI) is one of the most common causes of disability world-wide, leading to loss of productivity in the young, loss of quality of life, as well as a significant financial and social burden [1,2,3,4]. Switzerland has one of the highest age-adjusted number of deaths from TBI across Europe, indicating a high local impact [5,6]. Secondary injuries constitute pathophysiological brain insults in addition to the initial insult and may occur before arrival to hospital. Even a single episode of hypotension, hypoxia, or hyperventilation can worsen the initial brain injury, increasing morbidity and mortality [7,8,9,10,11,12]. Monitoring and stabilisation of the blood pressure is crucial in prehospital care, especially as the impairment of cerebrovascular autoregulation after TBI results in an overreliance on the systemic blood pressure for an adequate cerebral perfusion pressure [13]. Acceptable lower thresholds for the systolic blood pressure (SBP) for the management of patients with TBI in adults have evolved over time [14,15,16,17,18,19]. Current prehospital guidelines for the management of TBI recommend reaching a minimum target SBP threshold > 110 mmHg [20].

Hypertension equally causes harm in a patient with impaired cerebral autoregulation through cerebral hyperperfusion. Consequently, a raised intracranial pressure, haemorrhage, and cerebral oedema cause secondary injury. It is recognised that hypertension after TBI may also occur secondary to an excess of catecholamine [21,22]. Catecholamines have been shown to have neurotoxic effects, and the current guidelines recommend volume resuscitation to reach SBP targets [20,23]. Furthermore, SBP variability has been linked to poorer outcomes in TBI, but little evidence exists about SBP variability in the prehospital phase [24]. Based on several studies in the prehospital setting demonstrating increased mortality in hypertensive TBI patients, the acceptable upper limit of SBP has been defined, for the first time, by the newest guidelines as 150 mmHg [15,20,21,25,26,27]. Targeting the maladaptive mechanisms leading to hypertension may be promising as there is growing evidence for early exposure to beta-blockers improving the outcome after TBI [28,29]. Current guidelines recommend primarily close monitoring, rather than specific pharmacotherapy, implicating a risk of iatrogenic cerebral hypoperfusion in patients with an adaptive Cushing’s response [20].

In this retrospective cohort study, we aim to characterise the prehospital haemodynamic profile of patients requiring intensive care after TBI by integrating the newest recommendations for optimal SBP thresholds. Secondly, we aim to determine the profile’s impact on the outcome.

## 2. Methods

### 2.1. Study Design

In this single-centre, retrospective, registry-based observational study conducted at the Institute for Intensive Care (ICU), University Hospital of Zurich (USZ), Switzerland, all consecutive TBI patients, regardless of severity, admitted to the ICU between January 2018 and December 2022 were screened for eligibility. The local ethics committee approved this study. This study was performed in accordance with the ethical standards as laid out in the 1954 Declaration of Helsinki and its later amendments. Inclusion criteria were adults (≥18 years) diagnosed with TBI requiring ICU care and admitted to USZ within 24 h after trauma. Patients were excluded if they refused analysis of their data (documented written or oral refusal).

### 2.2. Prehospital Management

Prehospital management was exclusively conducted by the local emergency medical services (EMS) teams. Depending on triage by the EMS departments, teams were com-posed of either paramedics alone or paramedics in attendance of an emergency physician. Emergency physician dispatch in the area of Zurich is mandatory for entrapment or burial, trauma with loss of consciousness, severe head or neck injuries, electrical injuries, major burns (>30% TBSA), gunshot or stab wounds, limb amputations, severe chest trauma, or uncontrolled major bleeding. Local prehospital standard procedures are based on international guidelines.

### 2.3. Data Collection

Data was obtained retrospectively from EMS protocols, the electronic medical records (KISIM-TM; Cistec^®^, Zurich, Switzerland), and the electronic Patient Data Management System (MetaVision Suite; iMDsoft^®^, Tel Aviv, Israel). Data was managed using the Research Electronic Data Capture (REDCap v14.5.1; Vanderbilt 100 University, Nashville, TN, USA) [30,31]. Demographic baseline data collected includes age, sex, comorbidities based on the Charlson Comorbidity Index (CCI) [32], prior harmful alcohol or substance misuse, prior medication including antihypertensives, anticoagulation or antiplatelet therapy, clinical frailty index (CFI) [33], living situation and level of social or physical dependence, cause of trauma, and injury mechanism. The severity of the injury was assessed using the Injury Severity Score (ISS) [34] and Abbreviated Injury Score (AIS) [35], as well as the initial Glasgow Coma Scales score (GCS) [36,37].

Prehospital data: Time stamps on the EMS chart were used to calculate prehospital times. Vital parameters were collected from both handwritten and electronic charts where available, and laboratory values referring to the prehospital setting were obtained from the first arterial blood gas analysis after arrival in the emergency department (ED). In addition to absolute systolic blood pressure values (first, highest, and lowest prehospital as well the admission SBP at the ED), the range of SBP maintained for two-thirds of the prehospital time was considered. The upper limit of this range was used for statistical analysis, and the magnitude of this range was used as a representation of SBP variation. Furthermore, details about prehospital therapy including intravenous fluids, catecholamines, analgesia, intubation, and ventilation were collected. In-hospital mortality was obtained from the electronic patient records. The neurological functional outcome of patients at discharge was assessed with the Glasgow Outcome Score Extended (GOSE) [38].

### 2.4. Data Analysis and Statistics

We present descriptive qualitative statistics with absolute numbers and the corresponding percentages, whereas quantitative data are presented with the median and the IQR expressed as (Q1–Q3). Systolic blood pressures were grouped into hypotensive, normotensive, and hypertensive according to the ranges defined in the current prehospital guidelines (<110 mmHg, 110–149 mmHg, and ≥150 mmHg, respectively) [20]. GOSE was dichotomised into favourable and unfavourable outcome with groups defined as GOSE of ≥5 and <5, respectively. We compared the different random variables according to the survival and dichotomised GOSE with the Mann–Whitney test, the chi-squared test, or the Fisher exact test as appropriate. To identify potential predictors of in-hospital survival and of dichotomised GOSE at hospital discharge, we first performed a univariate logistic regression analysis, followed by a multivariable logistic regression model using variables with a *p*-value < 0.1 identified in the univariate analysis. We performed all statistical tests two-sided, and a *p*-value < 0.05 was considered statistically significant. We used the Stata version 17.0 software (StataCorp LP, College Station, TX, USA) for all statistical analyses.

## 3. Results

### 3.1. Demographics and Injury Mechanism—Table 1

Of the 680 included patients, the median age was 56 years (IQR 36–73). Patients were predominantly male (74%), with a good functional status pre-injury as indicated by the CCI and CFS (Table 1). Most patients had suffered major trauma, with a median ISS of 22 (IQR 14–29). Furthermore, 72% had moderate–severe TBI (AIS Head and Neck ≥3). The most common injury mechanism was a fall (52%), followed by a road traffic accident (37%).

**Table 1:** Patient demographics and injury severity.

Demographics	Total N = 680	Dead N = 117 (17.2%)	Alive N = 563 (82.8%)	*p*-Value	GOSE < 5 N = 473	GOSE ≥ 5 N = 207	*p*-Value
Age (years)	56 (36–73)	73 (55–83)	53 (35–71)	<0.001 *	58 (37–76)	50 (32–63)	<0.001 *
Male sex	503 (74%)	72 (62%)	431 (77%)	0.001 *	335 (71%)	168 (81%)	0.005 *
Arterial hypertension	177 (26%)	36 (31%)	141 (25%)	0.199	129 (27%)	48 (23%)	0.264
Antihypertensives	175 (26%)	48 (41%)	127 (23%)	<0.001 *	127 (27%)	30 (14%)	<0.001 *
Charlson Comorbidity Index	0 (0–1)	1 (0–3)	0 (0–1)	<0.001 *	0 (0–1)	0 (0–1)	<0.001 *
Clinical frailty index	2 (2–3)	3 (2–4)	2 (2–3)	<0.001 *	2 (2–4)	2 (2–3)	0.008 *
Injury Severity Score	22 (14–29)	29 (25–38)	21 (14–27)	<0.001 *	25 (17–33)	16 (10–22)	<0.001 *
AIS Head and Neck	3 (2–4)	5 (4–5)	3 (2–4)	<0.001 *	4 (3–5)	3 (2–3)	<0.001 *
AIS Chest	0 (0–3)	0 (0–3)	0 (0–3)	0.150	1 (0–3)	0 (0–3)	0.003 *
AIS Abdomen	0 (0–0)	0 (0–0)	0 (0–1)	0.001 *	0 (0–2)	0 (0–0)	0.016 *
Isolated TBI	213 (31.0%)	49 (42%)	164 (29%)	0.007 *	133 (28%)	80 (39%)	0.007 *

Patient demographics and injury severity. Values expressed as counts with percentages or median values with the interquartile range (Q1–Q3), as applicable. Statistical analysis of survival and dichotomised GOSE at hospital discharge. * Asterix highlights statistically significant values of *p*, as defined as *p* < 0.05. Abbreviations: AIS: Abbreviated Injury Score, GOSE: Glasgow Outcome Score Extended, ISS: Injury Severity Score, TBI: traumatic brain injury.

In total, 117 patients (17%) died. The functional outcome according to the GOSE was unfavourable (<5) in 473 patients (69.6%). Non-survivors were significantly older than survivors (median 73 [IQR 55–83] vs. 53 [35–71] years, *p* < 0.001). The male sex was less frequent among non-survivors (62% vs. 77%, *p* = 0.001). Non-survivors presented with a higher CCI (1 [0–3] vs. 0 [0–1], *p* < 0.001) and higher CFI (3 [2–4] vs. 2 [2–3], *p* < 0.001). The ISS and AIS Head/Neck were significantly higher in non-survivors (ISS 29 [25–38] vs. 21 [14–27], AIS 5 [4–5] vs. 3 [2–4], both *p* < 0.001). Isolated TBI was more prevalent among non-survivors (42% vs. 29%, *p* = 0.007).

### 3.2. Prehospital Assessment and Transport—Table 2

In 72% of our cases, a doctor was present at the site of injury, with a median transport time of 19 min and 30% transported by helicopter. The median GCS was 13 (IQR 7–14), with 20% showing a secondary worsening during the prehospital phase.

**Table 2:** Prehospital presentation and management.

Prehospital Presentation and Management	Total N = 680	Dead N = 118 (16.9%)	Alive N = 581 (83.1%)	*p*-Value	GOSE < 5 N = 473	GOSE ≥ 5 N =207	*p*-Value
Time from trauma until ED arrival [minutes]	78 (60–115)	85 (65–116)	75 (59–115)	0.027 *	80 (61–120)	70 (55–100)	<0.001 *
Air medical service	201 (30%)	34 (29%)	167 (30%)	0.94	151 (33%)	50 (26%)	0.057
Initial GCS	13 (7–14)	4 (3–11)	13 (9–15)	<0.001 *	12 (6–14)	14 (9–15)	0.002 *
Secondary GCS worsening	134 (20%)	26 (23%)	108 (19%)	0.395	104 (23%)	30 (15%)	0.019 *
Both pupils reacting	539 (79%)	54 (46%)	485 (86%)	<0.001 *	358 (81%)	181 (93%)	<0.001 *
Pupil asymmetry	101 (16%)	46 (41.1%)	56 (9.6%)	<0.001 *	87 (20%)	14 (7%)	<0.001 *
First SpO_2_	96 (90–98)	95 (89–99)	96 (91–98)	0.4854	95 (90–98)	96 (94–99)	<0.001 *
Lowest SpO_2_	93 (85–97)	92 (80–97)	93 (86–97)	0.051	92 (83–97)	94 (89–97)	0.003 *
Lowest blood glucose [mmol/L]	7.3 (6.1–9.0)	8.8 (7.1–10.6)	6.9 (6.0–8.6)	<0.001 *	7.7 (6.3–9.6)	6.3 (5.6–7.9)	<0.001 *
First measured haemoglobin [g/L]	12.4 (10.6–13.7)	10.7 (9.2–12.6)	12.6 (11.1–13.8)	<0.001 *	11.9 (10.1–13.3)	13.4 (12.1–14.4)	<0.001 *
Lowest body temperature [°C]	36.2 (35.7–36.7)	36 (35.1–36.6)	36.3 (35.8–36.7)	0.0015 *	36.2 (35.6–36.6)	36.4 (36–36.8)	<0.001 *
Prehospital intubation	271 (40%)	80 (70%)	191 (34%)	<0.001 *	207 (44%)	64 (31%)	0.01 *
Lowest etCO_2_ [kPa]	4.1 (3.5–4.6)	3.9 (3.0–4.5)	4.2 (3.6–4.7)	0.064	4.1 (3.3–4.6)	4.3 (3.8–4.8)	0.202
Highest etCO_2_ [kPa]	5 (4.4–5.73)	5.1 (4.3–5.9)	5.0 (4.5–5.7)	0.82	5.0 (4.4–5.7)	5.1 (4.4–5.7)	
Amount of intravenous crystalloids [mL]	300 (50–500)	450 (100–550)	300 (50–500)	0.2286	400 (100–600)	150 (0–500)	<0.001 *
Analgosedation received	474 (70%)	87 (74%)	387 (69%)	0.229	333 (70%)	141 (68%)	0.551
Catecholamines received	159 (23%)	48 (41%)	111 (20%)	<0.001 *	125 (26%)	34 (18%)	0.005 *

Prehospital presentation and management: Values expressed as counts with percentages or median values with the interquartile range (Q1–Q3), as applicable. * Asterix highlights statistically significant values of *p*, as defined as *p* < 0.05. Abbreviations: ED: emergency department, EMS: emergency medical services, etCO_2_: end-tidal carbon dioxide, GCS: Glasgow Coma Scale, GOSE: Glasgow Outcome Score Extended, ISS: Injury Severity Score, SpO_2_: peripheral oxygen saturation.

The median time from trauma to emergency department arrival was 13% longer in patients who died (85 [65–116] vs. 75 [59–115] minutes, *p* = 0.027). In comparison to surviving patients, patients who died had lower initial GCS scores (4 [3–11] vs. 13 [9–15], *p* < 0.001), more frequent pupil asymmetry (41% vs. 10%, *p* < 0.001), and non-reactive pupils (54% vs. 14%, *p* < 0.001), respectively. Patients who died were more frequently intubated prehospital (70% vs. 34%, *p* < 0.001) and treated with catecholamines (41% vs. 20%, *p* < 0.001) compared to surviving patients.

### 3.3. Prehospital Blood Pressure Profile Characterisation—Table 3

The median first recorded prehospital SBP was 135 mmHg, with 32% in the hypertensive and 19% in the hypotensive range. The median lowest SBP during the prehospital phase was 117 mmHg, with 39% of patients in the hypotensive group. Almost half of patients (47%) had a highest SBP ≥ 150 mmHg. The median SBP in the ED was 130 mmHg, with 51% of patients in the range of 110–149 mmHg and equal groups (24%) above and below this range. The median SBP during the majority (two-thirds) of the prehospital time was 140 mmHg, with a median variation of 15 mmHg. Most patients (65%) had an SBP variation ≤ 20 mmHg, while 11% had a variation > 40 mmHg.

**Table 3:** Prehospital systolic blood pressure characterisation.

Prehospital SBP Characterisation	Total N = 680	Dead N = 118 (16.9%)	Alive N = 581 (83.1%)	*p*-Value	GOSE < 5 N = 473 (69.6%)	GOSE ≥ 5 N = 207 (30.4%)	*p*-Value
First SBP [mmHg (IQR)]	135 (115–154)	143.5 (115–178)	135 (115–151)	-	135 (111–153)	137 (120–155)	-
Available *n* = 584							
<110 mmHg	111 (19%)	21 (21%)	90 (19%)		89 (22%)	22 (13%)	0.03 *
110–149 mmHg	285 (49%)	35 (34%)	250 (52%)	0.003 *	190 (46%)	95 (55%)	
≥150 mmHg	188 (32%)	46 (45%)	142 (29%)		132 (32%)	56 (32%)	
Lowest SBP [mmHg (IQR)]	117 (100–133)	113.5 (95–137)	119 (100–133)	-	115 (95–132)	120 (105–140)	-
Available *n* = 578							
<110 mmHg	224 (39%)	47 (46%)	177 (37%)	0.061	174 (43%)	50 (29%)	0.009 *
110–149 mmHg	282 (49%)	39 (38%)	243 (51%)		185 (45%)	97 (57%)	
≥150 mmHg	72 (13%)	16 (16%)	56 (12%)		49 (12%)	23 (14%)	
Highest SBP [mmHg (IQR)]	147 (128–167)	158 (130–189)	145 (127–162)	-	147 (125–170)	147 (130–164)	-
Available *n* = 578							
<150 mmHg	306 (53%)	40 (39%)	266 (56%)	0.002 *	216 (53%)	90 (53%)	1
≥150 mmHg	272 (47%)	62 (61%)	210 (44%)		192 (47%)	80 (47%)	
SBP range 2/3 prehospital time [mmHg (IQR)]	140 (120–160)	150 (120–174)	140 (121–158)	-	140 (120–160)	144 (125–160)	-
Available *n* = 576							
<110 mmHg	62 (11%)	12 (12%)	50 (11%)		55 (14%)	7 (4%)	<0.001 *
110–149 mmHg	286 (50%)	38 (37%)	248 (52%)	0.015 *	190 (47%)	96 (57%)	
≥150 mmHg	228 (40%)	52 (51%)	176 (37%)		162 (40%)	66 (39%)	
SBP variability [mmHg (IQR))]	15 (8–28)	22 (10–40)	15 (7–25)	-	17 (9–28)	13 (6–26)	-
Available *n* = 576							
0–20 mmHg	372 (65%)	50 (49%)	322 (68%)	<0.001 * with >40 mmHg	258 (63%)	114 (67%)	0.616
21–40 mmHg	141 (25%)	31 30%)	110 (23%)		102 (25%)	39 (23%)	
>40 mmHg	63 (11%)	21 (21%)	42 (9%)		47 (12%)	16 (9%)	
SBP at admission in ED [mmHg (IQR)]	130 (110–149)	124 (105–147)	130 (110–149)	-	128 (105–146)	130 (114–150)	-
Available *n* = 671							
<110 mmHg	162 (24%)	32 (28%)	130 (23%)	0.061	126 (27%)	36 (18%)	0.126
110–149 mmHg	345 (51%)	56 (48%)	289 (52%)		233 (50%)	112 (55%)	
≥150 mmHg	164 (24%)	28 (24%)	136 (25%)		110 (23%)	54 (27%)	

Prehospital SBP characterisation. Values are stated as counts with percentages or median values with the interquartile range (Q1–Q3), as applicable. Table includes statistically significant SBP group differences, as analysed with Pearson chi-squared test, followed by Fisher’s exact test. GOSE was dichotomised into favourable (≥5) and unfavourable (<5). * Asterix highlights statistically significant values of *p*, as defined as *p* < 0.05. Abbreviations: ED: emergency department, GOSE: Glasgow Outcome Score Extended, SBP: systolic blood pressure.

The first recorded SBP was higher in non-survivors (144 [115–178] vs. 135 [115–151] mmHg), with a larger proportion presenting with an SBP ≥ 150 mmHg (45% vs. 29%, *p* = 0.003). The lowest SBP was <110 mmHg in 46% of non-survivors versus 37% of survivors. SBP variability was significantly higher among non-survivors (22 [10–40] vs. 15 [7–25] mmHg), with a significantly higher proportion of non-survivors with a variability > 40 mmHg (21 vs. 9%, *p* < 0.001, compared to variability 0–20 mmHg). When analysing SBP over two-thirds of the prehospital time, nearly half of all patients (50%) remained within the target range (110–149 mmHg). This proportion was higher among patients with a favourable outcome (57% vs. 46%). A significantly higher proportion of non-survivors were in the hypotensive group (15% vs. 4%, *p* = 0.015).

### 3.4. Logistic Regression Analysis—Table 4 and Table 5

In univariate logistic regression, a younger age, male sex, higher initial GCS, higher first haemoglobin, and lower AIS Head/Neck and ISS were associated with better survival (all *p* < 0.001). Blood pressure variables (first SBP, SBP variability, SBP range) were significant in the univariate analysis but lost statistical significance in the multivariable model.

**Table 4:** Logistic regression for survival at hospital discharge.

Logistic Regression for Survival	Univariate Logistic Regression		Multivariable Logistic Regression	
	OR (95% CI) Survival	*p*-Value	OR (95% CI) Survival	*p*-Value
Age (year)	0.96 (0.95–0.97)	<0.001 *	0.95 (0.93–0.97)	<0.001 *
Sex (female)	0.49 (0.32–0.75)	0.001 *	1.10 (0.53–2.30)	0.798
AIS Head and Neck	0.27 (0.21–0.34)	<0.001 *	0.45 (0.29–0.70)	<0.001 *
Prehospital time (10 min)	0.99 (0.98–1.00)	0.037 *	0.99 (0.97–1.02)	0.608
Clinical Frailty Score	0.70 (0.60–0.81)	<0.001 *	0.76 (0.57–1.01)	0.055
First SBP (mmHg)	0.99 (0.98–1.00)	0.003 *	1.00 (0.99–1.02)	0.556
First SpO_2_ (%)	1.02 (1.00–1.04)	0.058	1.01 (0.98–1.05)	0.498
Amount of iv. Fluids (100 mL)	0.98 (0.95–1.02)	0.439	1.02 (0.95–1.09)	0.588
SBP variability (mmHg)	0.98 (0.97–0.99)	<0.001 *	1.01 (0.99–1.04)	0.194
SBP Range over 2/3rds of prehospital time (mmHg)	0.99 (0.98–1.00)	0.003 *	0.99 (0.97–1.01)	0.310
Administration of catecholamines	0.33 (0.21–0.51)	<0.001 *	0.72 (0.33–1.60)	0.421
Initial GCS	1.25 (1.19–1.31)	<0.001 *	1.24 (1.15–1.35)	<0.001 *
FO_2_Hb (per 0.1)	1.25 (0.95–1.64)	0.117	1.26 (0.69–2.32)	0.453
First haemoglobin (g/dL)	1.29 (1.19–1.40)	<0.001 *	1.24 (1.04–1.46)	0.014 *
Injury Severity Score	0.94 (0.92–0.95)	<0.001 *	0.96 (0.92–1.00)	0.053
AIS Chest	1.10 (0.97–1.25)	0.124	1.05 (0.80–1.38)	0.711
AIS Abdomen	1.07 (0.91–1.26)	0.419	0.99 (0.71–1.37)	0.941

Logistic regression analysis of prehospital parameters with survival at hospital discharge. Statistical analysis (univariate and multivariable logistic regression) includes the odds ratio (OR) of survival at hospital discharge with 95% confidence intervals. * Asterix highlights statistically significant values of *p*, as defined as *p* < 0.05. Abbreviations: AIS: Abbreviated Injury Score, SpO_2_: peripheral oxygen saturation, EMS: emergency medical services, FO_2_Hb Fraction of oxyhaemoglobin, GCS: Glasgow Coma Scale, OR: odds ratio, SBP: systolic blood pressure, SpO_2_: peripheral oxygen saturation.

**Table 5:** Logistic regression for GOSE at hospital discharge.

Logistic Regression for GOSE at Hospital Discharge	Univariate Logistic Regression		Multivariable Logistic Regression	
	OR (95% CI) Unfavourable GOSE (<5)	*p*-Value	OR (95% CI) Unfavourable GOSE (<5)	*p*-Value
Age (year)	1.02 (1.01–1.02)	<0.001 *	1.02 (1.01–1.04)	0.002 *
Sex (female)	1.77 (1.19–2.65)	0.005 *	0.96 (0.52–1.76)	0.895
AIS Head and Neck	1.47 (1.30–1.67)	<0.001 *	0.86 (0.64–1.16)	0.334
Prehospital time (10 min)	1.01 (1.00–1.02)	0.067	1.01 (0.99–1.03)	0.267
Clinical Frailty Score	1.23 (1.07–1.42)	0.003 *	1.09 (0.87–1.37)	0.455
First SBP (mmHg)	1.00 (0.99–1.00)	0.242	1.00 (0.98–1.01)	0.527
First SpO_2_ (%)	0.93 (0.91–0.96)	<0.001 *	0.96 (0.92–1.00)	0.032 *
Amount of iv. fluids (100 mL)	1.09 (1.05–1.11)	<0.001 *	1.08 (1.01–1.14)	0.018 *
SBP variability (mmHg)	1.01 (1.00–1.02)	0.168	1.00 (0.98–1.01)	0.883
SBP range over 2/3 of prehospital time (mmHg)	1.00 (0.99–1.00)	0.128	1.00 (0.98–1.02)	0.842
Administration of catecholamines	1.84 (1.20–2.81)	0.005 *	0.52 (0.28–0.98)	0.043 *
Initial GCS (g/dL)	0.94 (0.90–0.97)	0.001 *	0.96 (0.91–1.02)	0.229
FO_2_Hb (per 0.1)	1.15 (0.88–1.49)	0.309	1.38 (0.89–2.14)	0.154
First haemoglobin (g/dL)	0.72 (0.66–0.78)	<0.001 *	0.85 (0.75–0.97)	0.016 *
Injury Severity Score	1.10 (1.08–1.13)	<0.001 *	1.14 (1.08–1.20)	<0.001 *
AIS Chest	1.21 (1.09–1.35)	<0.001 *	0.72 (0.57–0.91)	0.005 *
AIS Abdomen	1.22 (1.06–1.41)	0.007	0.88 (0.67–1.14)	0.318

Logistic regression of prehospital parameters with GOSE at hospital discharge. GOSE was dichotomised into favourable (≥5) and unfavourable (<5). Statistical analysis (univariate and multivariable logistic regression) includes the odds ratio (OR) of unfavourable GOSE with 95% confidence intervals (CIs). * Asterix highlights statistically significant values of *p*, as defined as *p* < 0.05. Abbreviations: AIS: Abbreviated Injury Score, SpO_2_: peripheral oxygen saturation, EMS: emergency medical services, FO_2_Hb: fraction of oxyhaemoglobin, GCS: Glasgow Coma Scale, OR: odds ratio, SBP: systolic blood pressure, SpO_2_: peripheral oxygen saturation.

Catecholamine administration was also strongly associated with the outcome. In the univariate analysis, catecholamine use was linked to increased mortality and a higher likelihood of an unfavourable GOSE (<5) (OR 1.84 [95% CI: 1.20–2.81], *p* = 0.005). However, after adjusting for confounders with the multivariable model, catecholamine administration was no longer associated with mortality and was inversely associated with an unfavourable GOSE (OR 0.52 [0.28–0.98], *p* = 0.043), indicating a shift towards an improved functional outcome after adjustment.

No significant association was observed in the univariate model concerning physician attendance for either survival (OR 0.7 [0.4–1.2], *p* = 0.19) or an unfavourable GOSE (<5) (OR 1.44 [0.96–2.17], *p* = 0.075). Analgosedation was also not significantly associated with survival (OR 0.76 [0.48–1.19], *p* = 0.23) or an unfavourable GOSE (<5) (OR1.11 [0.78–1.58], *p* = 0.56).

In the final multivariable model for survival, independent predictors were a younger age (OR 0.95 [95% CI 0.93–0.97], *p* < 0.001), lower AIS Head/Neck (OR 0.45 [0.29–0.70], *p* < 0.001), higher initial GCS (OR 1.24 [1.15–1.35], *p* < 0.001), and higher first haemoglobin (OR 1.24 [1.04–1.46], *p* = 0.014).

For the functional outcome, independent predictors of an unfavourable GOSE (<5) included an older age (OR 1.02 [1.01–1.04], *p* = 0.002), lower initial SpO_2_ (OR 0.96 [0.92–1.00], *p* = 0.032), greater amount of intravenous fluids administered (OR 1.00 [1.00–1.00], *p* = 0.018), catecholamine administration (OR 0.52 [0.28–0.98], *p* = 0.043), lower first haemoglobin (OR 0.85 [0.75–0.97], *p* = 0.016), and higher ISS (OR 1.14 [1.08–1.20], *p* < 0.001). AIS Chest was inversely associated with an unfavourable outcome (OR 0.72 [0.57–0.91], *p* = 0.005).

## 4. Discussion

This retrospective cohort study provides a comprehensive profile of prehospital parameters and prehospital SBP of patients following TBI. As a main finding, we report that half of patients are within the recommended normotensive range at first assessment; however, the majority of patients experience SBP derangement outside the currently recommended range, placing them at risk of secondary injury during the prehospital phase. As another main finding, we report that after adjustment for confounders via multivariable analysis, catecholamine administration was linked to an improved functional outcome, indicating a possible role of haemodynamic stabilisation in the prehospital setting. Thirdly, we confirmed that the initial GCS, ISS, AIS Head/Neck, and initial haemoglobin are independent predictors of the outcome in TBI.

The mortality of 17% was within reported ranges (12–27%) [15,39]. The pre-injury health status was good, as indicated by a low CFI and CCI. Interestingly, no independent association between CFI and either outcome was found in our study. Frailty is not universally reported in similar TBI studies, but has been shown to be a significant predictor of adverse outcomes in trauma patients for both mortality and postoperative complications [40]. Not all studies confirm this association; for example, in a study investigating trauma ICU patients, Jennings et al. found a similar mortality (18.6%) with a higher median CCI of 2 (1–4), which was not independently associated with the outcome [41]. Compared to a Swiss cohort of severe TBI patients, the median transport time was shorter in our cohort (50 vs. 19 min), which may reduce the probability of secondary injuries occurring and their measurable effects on the outcome [39].

SBP parameters (first, range, and variability) were not significantly associated with either outcome in the multivariable analysis, indicating that SBP alone did not predict the outcome in our study population. Current prehospital guidelines for the management of TBI specify a desirable range of 110–149 mmHg as an SBP target based on several large retrospective analyses including EPIC [20]. Since the publication of this guideline, Knack et al. confirmed the U-shaped mortality association, with an optimal range between 110 and 158 mmHg and SBP of 132 mmHg [42]. The median initial SBP of our study population was close to this optimal SBP. Half of the patients in our study were in this range at the first assessment and adhered to this range for two-thirds of the prehospital time. However, it follows that the other half of the patients are at risk of secondary injury, as they were outside this range for most of the prehospital time. An even greater proportion was outside of the range with single measurements. The minimal SBP in our study cohort is comparable to other studies, with Spaite et al. finding an average lowest SBP of 124 mmHg [15]. Both mentioned studies demonstrate significant associations with mortality and benefited from a notably larger sample size only including severe head injury (AIS ≥ 3). Further studies demonstrated that hypertensive SBP values, especially > 160 mmHg, were associated with greater odds of in-hospital death [25,27]. During the entire prehospital phase, hypertensive SBP extremes were more common than hypotensive extremes in our study (47 vs. 39%). This aligns with the relatively high median initial SBP (135 mmHg) of patients. There was a general drop in median SBP values over the prehospital time, with more hypotensive patients at the ED than on initial assessment (24 vs. 19%). This represents a risk of hypotensive secondary injury in a further 5% of patients. Previous studies investigating this difference found significantly increased mortality if SBPs were not corrected by hospital arrival [43]. The high proportion of catecholamine use and iv. fluid therapy signals the awareness of first responders to haemodynamic stabilisation. The impact of this is supported by the association of catecholamine use with an improved functional outcome. Available evidence for catecholamine use in TBI is scarce, with a lack of studies investigating catecholamine use in the prehospital phase; Hosomi et al. demonstrated increased mortality in the vasopressor group after adjustment in patients with severe TBI, and similarly Navpreet et al. demonstrated increased mortality with vasopressor use in hospital [44,45]. A study investigating resuscitation strategies in trauma found no association with mortality for the use of norepinephrine [46]. Recommendations concerning catecholamine administration and the type in TBI patients in the prehospital setting are currently lacking [47]. The use of catecholamines in addition to volume resuscitation in cases of severe hypotension should be weighed against the risk of exacerbating the catecholamine excess state to counteract cerebral hypoperfusion. Future research investigating the combined effects of volume administration and catecholamines may further the understanding of prehospital haemodynamic management.

Altogether, we confirm that most patients with TBI are at risk of secondary injury due to haemodynamic derangement in the prehospital phase, which highlights the relevance of stringently monitoring for a possible role of early intervention for hypotension to meet current guideline targets. Our study did not observe a harmful association of stabilisation with catecholamines.

Recommendations concerning pharmacotherapy are currently lacking for patients exceeding the recommended SBP range. A large proportion of patients in our study were hypertensive during the prehospital phase. Due to the lack of randomised clinical trials, it is unclear whether targeting hypertension pharmacologically after TBI is beneficial in the prehospital phase. Increased mortality has been observed in patients, especially with extremes of SBP > 180 mmHg [15]. Observational data on in-hospital use of beta-blockers has shown a positive association with the functional outcome [48]. The use of calcium channel blockers is not recommended in an unselected patient group, with recent evidence pointing towards harm in the early phases of TBI [49]. The risk of iatrogenic hypotension may prevent this approach from becoming feasible in the prehospital setting. Further research with randomised trials is required, identifying therapeutic approaches regarding haemodynamic monitoring and stabilisation, feasible in the prehospital phase.

There is a lack of available studies investigating SBP variability in the prehospital phase. Tran et al. found an increased range of in-hospital SBP values within the first 24 h to be associated with increased mortality [50]. Zhang et al. found a larger standard deviation of SBP of in-hospital measurements to be negatively associated with discharge home rates [51]. In patients with diffuse axonal injury, Ren et al. did not find an association of SBP variability with the outcome [52].

In this cohort of TBI patients, SBP variability was higher in patients with a worse outcome. This may be secondary to haemodynamic instability, an increased catecholaminergic drive, or aggressive correction through medical intervention. SBP variability on its own was not independently associated with the outcome.

### 4.1. Strengths

The strength of this study was the collection of several SBP measurements at multiple time points throughout the prehospital period, providing a comprehensive assessment of the haemodynamic trends, duration, and SBP variability prior to hospital admission. In addition to peaks and troughs, we also include a calculation for the average SBP range limited to two-thirds of the prehospital timespan. The traditional well-validated clinical scoring systems, including the GCS and AIS, independently predicted the outcome, supporting the representativeness and external validity of our study population. For a more accurate representation of the identified relationships, a further strength was the application of a multivariable analysis to eliminate known confounders such as sex, age, ISS, SpO_2_, and catecholamine use. A further strength of this study was the analysis of consecutive patients with minimal exclusion criteria, minimising selection bias and maximising the generalisability of our findings.

### 4.2. Limitations

This study is limited by the observational nature of the data, owing to the retrospective design. We recognise that our study may have limited power to demonstrate SBP associations with the outcome at the physiologically close cut-offs chosen. The sample size was comparable to other single-centre studies investigating secondary injuries [53,54] but was substantially smaller than recent multicentre registry-based studies determining SBP thresholds [15,25,26,42,55]. A comprehensive national registry including the prehospital parameters of interest, however, does not exist in Switzerland. Due to sample size limitations, including all significant prehospital variables in the multivariable logistic regression model risked overfitting and reducing the accuracy of the model. To address this limitation, a subset of parameters was selected for inclusion in the multivariable model based on their clinical relevance, statistical significance in univariate analysis, and potential for multicollinearity. Our analysis focuses on prehospital physiological derangement as a risk of secondary injury, which is inferred from available evidence but not correlated to imaging findings, readmission rates, or biochemical findings. Further insults that may add secondary injury during a hospital stay were not accounted for in the data analysis and may pose a confounding effect. A further limitation of this study is the lack of a standardised treatment algorithm concerning the prehospital management, allowing for a variation in the possible treatment administered by different EMS providers in Switzerland. Not all EMS services were accompanied by a doctor, which could limit therapeutic interventions such as intubation and appropriate administration of fluid and catecholamines. This could affect the effectiveness of haemodynamic stabilisation. Treating major bleeding with deliberate permissive hypotensive SBP targets is at the discretion of the EMS team, but they were not specifically accounted for in the data analysis. Similarly, other treatment interventions, such as different types of sedatives, analgesia, and osmotic agents, may confound the outcome results but were not included in the final analysis. Owing to the retrospective design of this study, we determined the functional status classified by the GOSE without a patient interview, though that is not as intended by the authors of the GOSE Score, which is a recognised limitation of this study.

## 5. Conclusions

Haemodynamic instability increasing the risk of secondary brain injury is common after TBI and represents a modifiable prehospital factor. In patients with traumatic brain injury, survival and the functional outcome were primarily determined by age, neurological status, injury severity, and haemoglobin levels at admission. Although blood pressure variables lost significance after adjustment, catecholamine administration was independently associated with improved functional recovery in multivariable models. This suggests a possible role of prehospital haemodynamic management, although causality cannot be inferred.

## Data Availability

The raw data supporting the conclusions of this article will be made available by the authors on request.

## Abbreviations

The following abbreviations are used in this manuscript:

AIS	Abbreviated Injury Score
CCI	Charlson Comorbidity Index
CFI	Clinical frailty index
CI	Confidence interval
ED	Emergency department
EMS	Emergency medical services
etCO_2_	End-tidal carbon dioxide
GCS	Glasgow Coma Scale
GOSE	Glasgow Outcome Scale Extended
ICU	Intensive care unit
IQR	Interquartile range
OR	Odds ratio
PaCO_2_	Partial pressure of arterial carbon dioxide
PaO_2_	Partial pressure of arterial oxygen
SBP	Systolic blood pressure
SpO_2_	Peripheral oxygen saturation
TBI	Traumatic brain injury
USZ	University Hospital Zurich
